# Polymer Coatings of Cochlear Implant Electrode Surface – An Option for Improving Electrode-Nerve-Interface by Blocking Fibroblast Overgrowth

**DOI:** 10.1371/journal.pone.0157710

**Published:** 2016-07-08

**Authors:** C. Hadler, P. Aliuos, G. Brandes, A. Warnecke, J. Bohlmann, W. Dempwolf, H. Menzel, T. Lenarz, G. Reuter, K. Wissel

**Affiliations:** 1 Department of Otorhinolaryngology, Hannover Medical School, Hannover, Germany; 2 Institute for Technical Chemistry, University of Technology Braunschweig, Braunschweig, Germany; 3 Cluster of Excellence “Hearing 4 All”, Hannover, Germany; 4 Institute of Cell Biology, Center of Anatomy, Hannover Medical School, Hannover, Germany; Institute for Frontier Medical Sciences, Kyoto University, JAPAN

## Abstract

Overgrowth of connective tissue and scar formation induced by the electrode array insertion increase the impedance and, thus, diminish the interactions between neural probes as like cochlear implants (CI) and the target tissue. Therefore, it is of great clinical interest to modify the carrier material of the electrodes to improve the electrode nerve interface for selective cell adhesion. On one side connective tissue growth needs to be reduced to avoid electrode array encapsulation, on the other side the carrier material should not compromise the interaction with neuronal cells. The present in vitro-study qualitatively and quantitatively characterises the interaction of fibroblasts, glial cells and spiral ganglion neurons (SGN) with ultrathin *poly(N*,*N-dimethylacrylamide)* (PDMAA), *poly(2-ethyloxazoline)* (PEtOx) and *poly([2-methacryloyloxy)ethyl]trimethylammoniumchlorid)* (PMTA) films immobilised onto glass surfaces using a photoreactive anchor layer. The layer thickness and hydrophilicity of the polymer films were characterised by ellipsometric and water contact angle measurement. Moreover the topography of the surfaces was investigated using atomic force microscopy (AFM). The neuronal and non-neuronal cells were dissociated from spiral ganglions of postnatal rats and cultivated for 48 h on top of the polymer coatings. Immunocytochemical staining of neuronal and intermediary filaments revealed that glial cells predominantly attached on PMTA films, but not on PDMAA and PEtOx monolayers. Hereby, strong survival rates and neurite outgrowth were only found on PMTA, whereas PDMAA and PEtOx coatings significantly reduced the SG neuron survival and neuritogenesis. As also shown by scanning electron microscopy (SEM) SGN strongly survived and retained their differentiated phenotype only on PMTA. In conclusion, survival and neuritogenesis of SGN may be associated with the extent of the glial cell growth. Since PMTA was the only of the polar polymers used in this study bearing a cationic charge, it can be assumed that this charge favours adhesion of both glial cells and SG neurons glial cells and SGN.

## Introduction

So far, the only therapeutic intervention for patients with profound sensory neural hearing loss is the chronic electrical stimulation of the residual auditory neurons via a cochlea implant (CI) [1–3]. However, insertion of the CI into the scala tympani evokes electrode insertion trauma resulting in mechanical damage of the lateral wall, basilar membrane and even the medial wall [4–5] as well as in inflammation and programmed cell death [6–7]. Moreover, fibrosis and new bone formation inside the scala tympani [8–11] and most adversely, growth of fibrous tissue on the implant surface [11–12] were found. In consequence, not only the impedance at the electrode–tissue interface increases [13–14] and higher power impact is needed to ensure CI performance, but also selective neuronal stimulation for discrimination between different sound frequencies is disturbed. Therefore, it is of great clinical interest to modify the surface of carrier material not only of auditory implants but also for other stimulating neural probes to inhibit connective tissue formation.

In general, adhesion of cells to various surfaces is mediated by secretion of fibrous proteins and various proteoglycans forming a complex extracellular matrix (ECM) allowing cell adhesion and providing biochemical and biomechanical signals for the control of behaviour and plasticity of the adhering cells [15–21]. However, various physiochemical properties like electrical charge, polarity and hydrophilicity/hydrophobicity-balance of the surface determine the adsorption of ECM components to the surfaces. Hereby, engineering and modification of the surface of artificial materials, which are used as medical implants, give great impact on cell and tissue interactions by the physical, biochemical and topographical properties of their surface [22–29].

For the design of cell selective implant surfaces in particular, thin films of polymers as like as *poly(N*,*N-dimethylacrylamide)* (PDMAA) [30–35] and *poly(2-ethyloxazoline)* (PEtOx) were found to be hydrophilic and protein repellent [32, 35–36]. Cell adhesion assays with another hydrophilic polymer, *poly(2-methacryloyloxy)ethyl-trimethylammoniumchlorid* (PMTA), revealed contradictory results. Depending on the cell type, PMTA was found either to inhibit or to enhance cell attachment. For example, Adden et al. [32] reported a significantly restricted growth of osteogenic precursor cells on PMTA films. By contrast, early studies presented PMTA as highly adhesive surface for the human endothelial cell line, Hep G2 (human liver carcinoma) as well as rat and sheep fibrocytes [31, 37–38]. Another study showed an increase in both protein adsorption and adhesion of MC3T3-E1 cells derived from newborn mouse calvaria with higher concentration of PMTA in the polyethylene glycol diacrylate (PEGDA) hydrogels [39]. Also, Rühe et al. [40] described good adhesion and neurite outgrowth of cerebellar neurons on *poly(2-methacryloyloxy)propyl-trimethylammoniumbromide* which differ from PMTA by a propyl-group.

Despite the differences in physicochemical features, biocompatibility of PDMAA [41–43], PEtOx [44–47] and PMTA [38, 39, 48–49] was demonstrated *in vitro* and *in vivo*, and, thus, these polymers may provide eligible coatings for medical implants.

So far, cell adhesion of primary neurons of the auditory nerve (spiral ganglion neurons, SGN) and non-neuronal cells (i.e. fibroblasts as well as glial cells) comprising Schwann and satellite glial cells on the polymers PDMAA, PEtOx and PMTA has not been characterised. Restriction of the formation of connective tissue around the carrier material of the electrode is desired for all neural probes and the expected benefits would apply for the improved electrical stimulation in neuroprostheses users suffering from Parkinson, neurodegenerative and psychiatric disorders, severe hearing loss or other sensorineural disabilities. Concurrently, nerve fiber growth towards the electrode contacts should be enabled.

In the present study, ultrathin polymer films were synthesised and immobilised on glass plates as described previously [35, 50]. The topography of the polymer monolayers was determined using atomic force microscopy (AFM). We characterised and quantified adhesion and growth of fibroblasts and glial cells, survival rate and neurite sprout of the SGN from postnatal rats on PDMAA, PEtOx and PMTA by immunocytochemical staining of cell specific neuronal and intermediary filaments as well as scanning electron microscopy.

## Materials and Methods

### Chemicals

Allyl bromide (99%), calcium hydride (CaH_2_) (95%), chlorodimethylsilane (98%), N,N-dimethylacrylamide (99%), 4-hydroxybenzophenone (98%), [2-(methacryloyloxy)ethyl]tri-methylammonium chloride solution, 80 wt% in H_2_O, Pt on activated charcoal (puriss. 10% Pt), Poly(2-ethyl-2-oxazoline) (PEtOx), sodium persulfate (98%) and triethylamine (99,5%) were purchased from Aldrich (St. Louis, USA). Azobisisobutyronitrile (AiBN) (98%) was obtained from Acros (Geel, Belgium). Potassium carbonate (99%), sodium hydroxide (99%) and sodium sulfate (99%) were purchased from Roth (Karlsruhe, Germany). Diethyl ether (HPLC grade) and toluene (HPLC grade) were obtained from VWR (Radnor, USA) and methanol (HPLC grade) from Fisher Scientific (Waltham, USA). Diethyl ether was dried over sodium and distilled before use. Triethylamine was dried over CaH_2_ and distilled before use. N,N-Dimethylacrylamide was distilled under vacuum before use.

### Polymer synthesis

#### Poly(dimethylacylamide) (PDMAA)

3 ml (29.11 mmol) N,N-dimethylacrylamide (DMAA) were dissolved in 26 ml methanol. Oxygen in the monomer solution was removed by purging with a moderate stream of nitrogen for 10 min. The polymerization was started by adding 48 mg (0.29 mmol) of 2,2′-Azobis(2-methylpropionitrile) (AIBN). The mixture was stirred for 1 h at 60°C. The polymerization was terminated by cooling the mixture to room temperature. 2.1 g of PDMAA were obtained (yield: 73%) after purification and isolation via dialysis against deionized water for 4 days and lyophilisation.

^1^H-NMR (D_2_O, 400 MHz, δ in ppm): 1.15–1.95 (m, 2H, C*H*_2_), 2.35–2.80 (m, 1H, C*H*), 2.80–3.20 (m, 6H, C*H*_3_).

FTIR (KBr, in cm^-1^): 2928 (*C-H*, alkyl), 1640 (*C = O*, *N*,*N*-disubstituted amide).

#### Poly([2-(methacryloyloxy)ethyl]trimethylammonium chloride) (PMTA)

An aqueous solution of [2-(methacryloyloxy)ethyl]trimethylammonium chloride (10 g; 80 wt.%, 38.52 mmol) were diluted with 38 ml deionized water to generate a monomer concentration of 1 mol/L in the reaction mixture. After purging with a moderate stream of nitrogen for 10 min, the polymerization was started by adding of 92 mg (0.39 mmol) sodium persulfate (Na_2_S_2_O_8_). The mixture was stirred for 1 h at 60°C. The polymerization was terminated by cooling the mixture to room temperature. For purification the polymer solution was dialyzed at first against a 0.1 M NaCl solution for 1 day and subsequently against deionized water for 4 days. After lyophilisation 7,1 g of PMTA were obtained (yield: 88,8%).

^1^H-NMR (D2O, 400 MHz, δ in ppm): 0.50–2.60 (m, 5H, C*H*_3_, C*H*_2_), 3.10–3.50 (s, 9H, C*H*_3_), 3.65–4.05 (m, 2H, C*H*_2_), 4.20–4.75 (m, 2H, C*H*_2_).

FTIR (KBr, in cm^-1^): 3018 (*C-H*, alkyl), 1729 (*C = O*, ester)

### Synthesis of the photochemically reactive silane anchor

The photoactive silane anchor 4-(3-chlorodimethylsilyl)propyloxybenzophenone was obtained by a standard two step-method according to Prucker et al., 1999 [50]. Briefly, in the first reaction step 4-allyloxybenzophenone is synthesized through a Williamson ether synthesis with 4-hydroxybenzophenone and allyl bromide. A subsequent hydrosilation of this reaction product with an excess of chlorodimethylsilane and platinum on charcoal (Pt-C, 10% Pt) as catalyst yields the benzophenone group containing silane anchor.

### Immobilization of the silane anchor onto glass plates

For the anchor immobilization and polymer coating glass plates (8 x 8 mm) were used. Prior to the immobilization process the glass plates were washed each with acetone, chloroform, methanol and deionized water three times for each solvent in an ultrasonic bath for 15 min (Sonorex RK 510, Bandelin electronic GmbH & Co KG, Berlin, Germany). The glass plates were treated with oxygen plasma for 30 min for further cleaning as well as generation of a high density of silanol groups on the glass surface using a low pressure plasma system (Femto series, Diener electronic GmbH + Co. KG, Ebhausen, Germany). The cleaned and activated glass plates were immersed into a 5 mM solution of the bifunctional silane anchor in toluene under dry conditions and nitrogen atmosphere for 24 h at room temperature. The solution of the silane anchor contains approximately 10 vol.% of triethylamine, which act as catalyst and as scavenger for the emerging HCl [50]. After immobilization of the silane anchor the glass plates were washed with chloroform three times in an ultrasonic bath for 5 min (Sonorex RK 510). The immobilization of the silane anchor was proven by determination of the wettability of the treated glass plates via static contact angle measurements. For investigation of the film thickness the silane anchor was also immobilized onto silicon plates by the same procedure. The thickness of the resulting anchor layer was subsequently determined by ellipsometry.

### Polymer coating of the glass plates

For the polymer coating the glass plates functionalized with the photochemically reactive silane anchor were covered with the polymers via spin coating and subsequently irradiated with UV-light. Details of these coating steps are described in our previous publication [35]. After irradiation the polymer coated glass plates were washed with methanol three times in an ultrasonic bath (Sonorex RK 510) for 10 min to remove unbounded polymer. The polymer coatings were characterized by determination of the wettability via static contact angle measurements and by ellipsometric investigation of the layer thickness of polymer coated silicon plates.

### Spectroscopic methods

#### NMR spectroscopy

The ^1^H-NMR spectra were recorded using the spectrometers AVIII 400 (Bruker, Billerica, USA) at a frequency of 400 MHz and with the magnetic field strength of 9.4 T.

#### FTIR spectroscopy

The IR spectra were obtained with the spectrometer Equinox 55 (Bruker). For the measurements KBr pellets were prepared containing 150 mg of KBr and 1.5–2.5 mg of the sample. Pellets from pure KBr were used as reference.

### Static contact angle measurements

For the static contact angle measurements, the sessile drop method and deionized water as liquid phase were used. The measurements were performed at room temperature and using the measuring instrument OCA 15 and the software SCA202 V.4.1.17 (DataPhysics Instruments GmbH, Filderstadt, Germany). All values presented in this study are means of 30 samples (n = 30).

### Ellipsometry

Polished silicon plates which have a glass like silicon oxide surface were used for the ellipsometric analysis of the layer thickness. The film thickness of the anchor layer and the polymer coating on the silicon plates was determined using a multiscope (Optrel GbR, Sinzing, Germany) in the ellipsometry mode with an incidence angle of 70°. All presented values are means of 10 samples (n = 10). For each sample 16 measuring points were recorded and the samples were measured before treatment for referencing and after anchor immobilization and polymer coating. The layer thicknesses were calculated by the software Elli (version 3.2., Optrel GbR).

### Morphological characterisation of the polymer surfaces by means of AFM

To characterize physical surface properties such as topography and surface roughness of the polymer coatings, a Nanowizard II AFM (JPK-Instruments AG, Berlin, Germany) was used. Glass slides with dimensions of 7.5 x 7.5 x 1 mm (length x width x height) were coated with PDMAA, PEtOx and PMTA as described above (cf. 2.5). For investigating their surface topography, polymer-coated glass slides were glued (reprorubber thin pour, Flexbar, USA) onto Petri dishes (TPP, Trasadingen, Switzerland) and were rinsed several times with PBS followed by several washing steps with distilled water. After the samples were dried the Petri dishes were installed onto a sample holder (Petri-dish heater, JPK-Instruments AG, Berlin, Germany) that was mounted above an inverted light microscope (AxioObserver A1, Zeiss, Jena, Germany). CSC37/NoAl cantilevers (μmash, Tallinn, Estonia) with nominal force constant of 0.8 N/m and resonance frequency of 40 kHz were mounted on AFM and sample surfaces were scanned in air in intermittent contact mode. To measure the average roughness (R_a_) of the polymer films, two different samples from each polymer coating were investigated each at four different scan fields (25 x 25 μm^2^) using a line rate of 0.5 Hz and a pixel number of 512 x 512. The measured values (n = 8) were averaged for each polymer type and are given in Table 1. By decreasing the scan field size to 5 x 5 μm^2^ and keeping the pixel number of 512 x 512, the resolution was enhanced in order to visualize the nanometric surface pattern of the polymer coatings at certain regions of interest within the surface areas which were scanned before. For processing the image data as well as for calculating the roughness (R_a_) values, the JPK SPM data processing software (JPK-Instruments AG, v. 4.3.25) was used.

**Table 1:** Average roughness (R_a_) values for polymer-coated and uncoated glass surfaces determined by means of AFM.

	Glass	PDMAA	PEtOx	PMTA
**Average roughness [nm], n = 8 scan areas, mean ± SD**	0.4 ± 0.17	0.4 ± 1.4[p < 0.01]	0.66 ± 0.14	0.62 ± 0.13

### Spiral ganglion (SG) neuron preparation and dissociation

Neonatal Spraque-Dawley rats (P3-5, n = 18 per experiment, n = 5 independent experiments) were used for SG dissection in accordance with the institutional guidelines for animal welfare of Hannover Medical School following the standards described by the German “Law on protecting animals” (Tierschutzgesetz). The mere killing of rats for tissue analysis is registered with the local authorities (Zentrales Tierlaboratorium, Hannover Medical School) and reported on a regular basis as demanded by law, but needs no further approval if no other treatment is applied before sacrifice (§4–106 2013/44).

After decapitation and removal of the skin, the skull was cut along the midline and bisected. The brain was removed and the halves of the two skull bases were immersed in ice-cold PBS (Invitrogen, Karlsruhe, Germany). Further dissection was performed under microscopic view (Leica MZ-6, Bensheim, Germany). The bony cochlear capsule was carefully opened to remove the stria vascularis and the organ of Corti from the modiolus. Finally, the entire SG was separated from the modiolus and placed in ice-cold Ca^2^+/ Mg^2^+-free Hank’s balanced salt solution (HBSS, Invitrogen, Darmstadt, Germany).

The enzymatic dissociation of SG containing not only the neurons, but also fibroblasts and glial cells, was performed as described previously by Berkingali et al, 2008 [51]: The SG were incubated in 2 ml Ca^2^+/Mg^2^+-free HBSS (Invitrogen) containing 0.1% trypsin (Serva, Heidelberg, Germany), 0.01% DNase I (Roche, Mannheim, Germany) and 0.1% collagenase (Sigma, St Louis, Missouri, USA) for 16–20 min at 37°C. The enzymatic activity was stopped by adding 200 μl FBS (Biochrom AG, Berlin, Germany). The supernatant was discarded and the cell clusters were washed three times in Panserin 401 (PAN Biotech GmbH, Aidenbach, Germany) supplemented with 25 mM HEPES (Invitrogen), 30 U/ml penicillin (Grünenthal, Aachen, Germany), 0.15% glucose (Braun, Melsungen, Germany), 8.75 μg/ml insulin (Sigma-Aldrich, Taufkirchen, Germany) and 1x N2-supplement (Invitrogen). The cell clusters were gently disrupted by pipetting up and down the suspension using 1000 μl and 200 μl filter tips (StarLab, Ahrensburg, Germany), respectively. The cell yield was determined by using the Neubauer cell count-chamber following staining of a small aliquot of the cell suspension with 10% trypan blue (Biochrom) to exclude apoptotic cells from the cell count.

### Seeding of the cells onto the glass plates coated with PDMAA, PEtOx and PMTA

Prior to the cell seeding, the glass plates (8x8 mm) with and without polymer coating were placed into the wells of a 48well-microtiter plate (Multiwell, Becton Dickinson Labware) and washed with 70% ethanol and PBS. Additionally, the glass plates without polymer used as positive control (PosCtrl) were coated with 0.1 mg/ml poly-DL-ornithine (Sigma) for 1 h at room temperature and 0.01 mg/ml laminin (Invitrogen) at 37°C for 1 h as described previously [51]. Following removal of the adhesion protein solutions the wells were rinsed with PBS (Biochrom).

The experimental setting was as follows: For each polymer 2 x 10^4^ dissociated cells were seeded in n = 5 wells supplied with a polymer coated glass plate. As positive control and reference for SGN survival the same cell number was seeded in n = 5 wells supplied with ornithine/laminin coated glass plates. Additionally, n = 5 glass plates without any coating were used as negative control. The cells were cultivated in 500 μl culture medium containing 10% FBS and the completed Panserin 401 as described above. The cultivation assay was repeated 5 times (N = 5) and prepared as described above to be cultivated at 37°C and 5% CO_2_ for 48 h in a humidified incubator followed by fixation with methanol.

### Immunocytochemical detection of cell specific antigens

To examine the composition of the population of the SG following cultivation on the polymers PDMAA, PEtOx and PMTA as well on glass controls, immunocytochemical staining of cell specific antigens was performed. For that, the cells were cultivated on the samples and fixed with methanol as described above. Tables 2 and 3 represent the primary and secondary antibodies used in this study. The cells were permeabilised with PBSTx (0.1% (w/v) Triton X-100 (Sigma-Aldrich) in PBS, pH 7.5, Biochrom AG) for 5 min and washed three times with PBS. The primary antibodies were diluted in PBS containing 1% bovine serum albumin (BSA, Sigma-Aldrich) as described in Table 2, and the samples were incubated for 1 h at room temperature. After washing with PBS three times for 5 min each the specific antigen-antibody interactions were detected by incubation with fluorescently labelled secondary antibodies (Table 3) diluted each 1:400 in 1% BSA/PBS for 1 h at room temperature in darkness. The specificity of the immune staining was verified by omission of the primary antibodies within the light exposure range between 1.5 and 2 sec. The washing steps were performed as described above. The nuclei of the cells was stained with DAPI (Prolong^®^ anti-fade Gold with DAPI, Invitrogen), diluted 1:300 in PBS. Positively stained antigens were visualised and analysed by fluorescence microscopy (Keyence BZ 9000 Biorevo, Keyence International, Mechelen, Belgium).

**Table 2:** Primary antibodies used in this study. Listing of the primary for immunostaining of cell specific antigens in the SGN, fibroblasts and glial cells.

Primary antibody	Host	Description	Specificity	Manufacturer	Dilution
Neurofilament 200 kD, monoclonal	Mouse	Intermediary filament	Neurons	Novocastra #NCL-NF200	1:400
P75, polyclonal	Rabbit	Neurotrophic growth factor receptor	Glial cells	Abcam #38335	1:500
Vimentin clone V9, monoclonal	Mouse	Intermediary filament	Fibroblasts, glial cells	Dako #M0725	1:200
Vimentin, polyclonal	Chicken	Intermediary filament	Fibroblasts, glial cells	Abcam #24525	1:750

**Table 3:** Secondary antibodies used in this study.

Secondary antibody IgG (H+L)	Host	Description	abs/em [nm]	Manufacturer
Anti-chicken	Goat	Texas Red (**GaCh TR**)	586/605	Santa Cruz Biotech. #sc2994
Anti-mouse	Goat	New Dylight 488 (**GaM 488**)	493/518	Jackson-Immunoresearch #115-485-008
Anti-rabbit	Goat	Alexa Fluor 594 (**GaRb 594**)	591/616	Jackson-Immunoresearch #111-515-144

Additionally, the number of positively stained fibroblasts (mouse anti-Vimentin antibody) and glial cells (rabbit anti-p75 antibody) adhering on the polymer films as well as on glass plates coated with ornithine/laminin assigned as positive control were determined in relation to the total number of DAPI stained cells. For that, n = 2–5 images each from n = 2 experiments were considered.

### Immunocytochemical determination of the survival rate and neurite outgrowth of the SGN

To determine the survival rate and neurite outgrowth of the SGN, immunocytochemistry was performed using monoclonal anti-mouse neurofilament-antibody (200 kD, clone RT97, Novocastra Ltd, Newcastle upon Tyne, UK) on methanol-fixed cells as described previously [52]. Briefly, the fixated SGN were incubated with a 1:500 dilution of the monoclonal mouse 200-kD anti-neurofilament antibody (Novocastra Ltd, Newcastle upon Tyne, UK) in 1.5% normal horse serum in PBS (Vectastain Elite ABC-Kit, Vector Labs, Burlingame, USA) for 1 h at 37°C, followed by rinsing with PBS three times prior to the incubation with the biotinylated anti-mouse IgG-antibody (Vector Labs) diluted 1:2000 in 1.5% normal horse serum in PBS for 30 min at room temperature. Following washing with PBS as described above, the fixed cells were incubated with the ABC complex solution (Vector Labs) for 30 min at room temperature according to manufacturer’s protocol. The labelling was visualized using peroxidase diaminobenzidine substrate (Peroxidase Substrate Kit DAB, Vector Labs) for 12 min, followed by washing with PBS.

The stained soma and neurites of the SGN were detected by using light microscopy (Zeiss Axio Observer Z1, Zeiss, Jena, Germany). Images were taken digitally by the CCD colour camera (Hitachi HV-D30, Hitachi Kokusai Electric, Japan) and processed by using the softwares Palm Robo (Palm Zeiss, München, Germany) and Analysis vs. 3.0 (SIS, Münster, Germany). All positively stained SGN were counted in each well for further data analysis. Neurites of at least three times the average cell diameter were included for data collection. Survival rates (in %) refer to the number of SGN in the experimental groups following 48 h of cultivation compared to the numbers of SGN adhering on glass plates without polymer. Neurite outgrowth was determined by measuring the lengths of 5 randomly selected neurites in each well of each group.

### Scanning electron microscopy

All cell types of the SG from neonatal rats cultivated on glass plates with and without polymer coatings for 48 h were rinsed in PBS and immediately fixed in 2.5% glutardialdehyde (Polysciences, Warrington, PA, U.S.A.) in 0.1 M sodium cacodylate (Merck, Bioscience, Darmstadt, D), pH 7,3, for 1h at room temperature. After dehydration in graded acetone (Mallinckrodt Baker R.V., Deventer, NL), the specimens were dried with CO_2_ in the CPD 030 (Balzers Union, Balzers, FL) mounted on aluminium holders, sputtered with a 20 nm thick gold layer and analyzed with SEM 505 (Philips, Eindhoven, NL). The micrographs were taken with SEM-Software as described by Gebert and Preiss, 1998 [53] and processed with Adobe Photoshop CS6.

### Statistical analysis

All data achieved from the *in vitro* cell culture assays were presented as mean ± standard error of mean (SEM). Mann-Whitney-test and one way nonparametric analysis of variance (ANOVA) and Newman-Keuls multiple comparison test were used for statistical assessment of the *in vitro* assays as noted in the result section. AFM data were presented as mean ± standard deviation (SD).

## Results

### Polymer coating of the glass plates

For the coating of glass plates with the hydrophilic polymers PDMAA, PEtOx and PMTA a method according to Prucker et al., 1999, was used [50]. At first, a benzophenone group containing silane anchor was immobilized onto the glass plates. Subsequently, the glass plates were spin coated with the respective polymer and irradiated with UV light resulting in a covalent attachment of the polymer. The successful immobilization of the silane anchor was confirmed by static contact angle (WCA) measurements and ellipsometric analysis of the layer thickness for silicon plates treated under the same conditions. As shown in Table 4, we found a rise of the WCA from 29° ± 3 measured on untreated glass plates to 68° ± 4 and a layer thickness of 1.2 nm ±0.3 following silane anchor immobilization. The polymer attachment was also proven by WCA measurements and ellipsometric investigation of the resulting layer thickness on polymer-treated silicon plates. Hereby, a substantial reduction of the water wettability was observed for all polymer samples in comparison to the WCA after immobilization of the silane anchor (PDMAA: 33° ± 1; PEtOx: 42° ± 1; PMTA: 16° ± 5). PDMAA and PEtOX showed layer thicknesses of about 11 nm ± 2 and 12 nm ± 2, respectively, whereas PMTA markedly yields a lower layer thickness of about 5 nm ± 1.

**Table 4:** Water contact angle (WCA) and layer thickness determined by ellipsometry.

	Glass	Silane anchor	PDMAA	PEtOx	PMTA
**Water contact angle [°]**	29 ± 3	68 ± 4	33 ± 1	42 ± 1	16 ± 5
**Layer thickness [nm]**	-	1.2 ± 0.3	11 ± 2	12 ± 2	5 ± 1

### Determination of the surface topography of the polymer coatings by AFM

**Fig 1:**
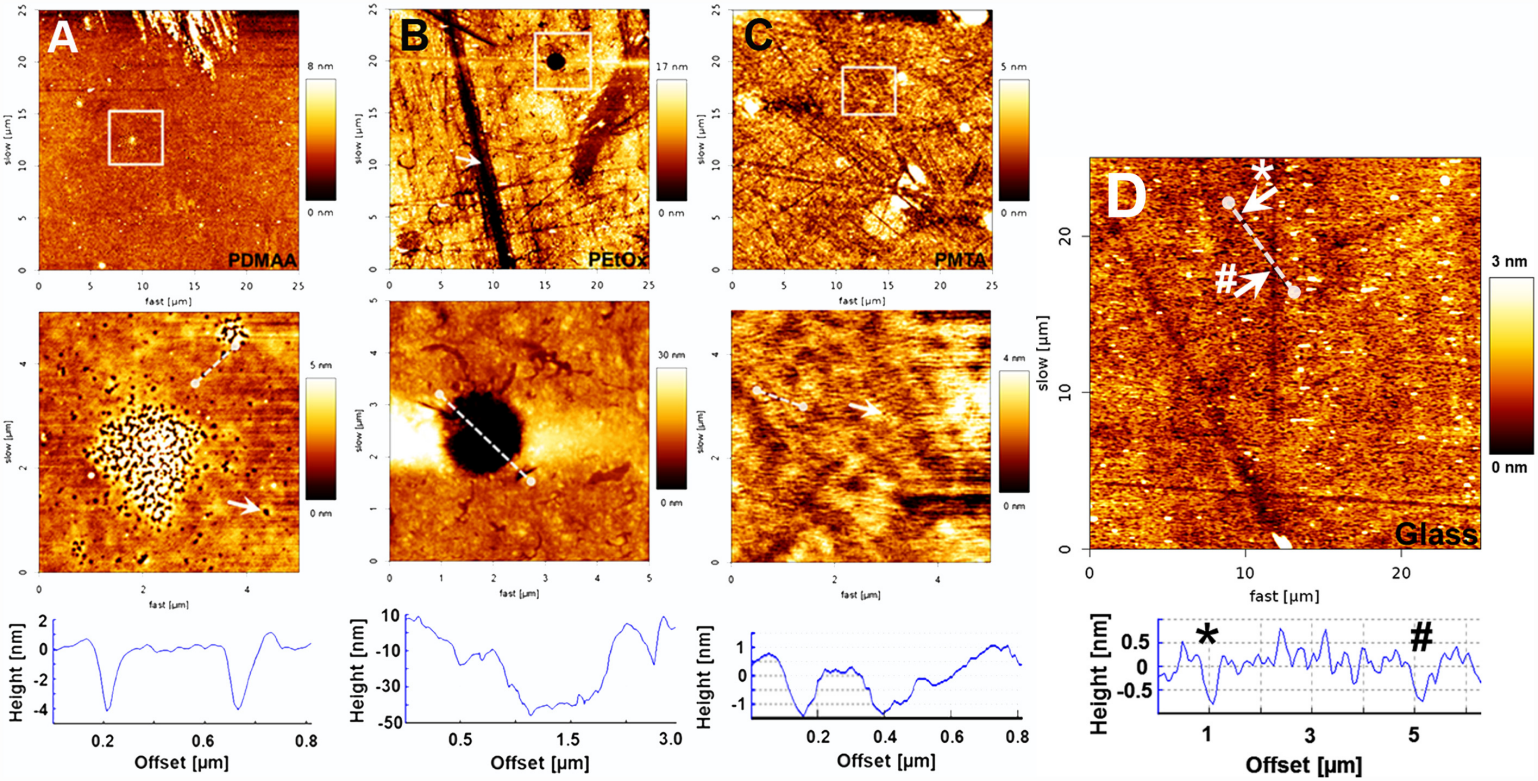
Representative AFM height images of polymer-coated and uncoated glass surfaces. Scan fields with a size of 25 x 25 μm were investigated by means of AFM on uncoated glass surfaces prior to immobilization of the silane anchor as control surface as well as on the polymer-coated glass plates to determine the impact of surface functionalizations on the surface roughness (A, B and C, upper row). Corresponding enlargements with enhanced resolution, indicated by the white squares in the upper row, are illustrated in the bottom row to visualize the nano-pattern of each surface type. (**A**) PDMAA coatings showed small deepenings with depths of approximately 4 nm and widths of about 0.1 μm (white arrow and cross section), whereas (**B**) PEtOx layers presented much larger hollows with diameters of approximately 2 μm and depths of around 50 nm. The white arrow points out channel-like deepenings with widths of around 2 μm and depths of 10–12 nm. (**C**) PMTA coated surfaces demonstrated lots of small deepenings with depths and widths of around 1.5–2.0 nm and 0.1 μm, respectively. (**D**) uncoated glass surfaces showed small indentations with depths of approximately 1 nm and width of about 0.5–1 μm (white arrows and cross section).

AFM was used to determine the topography and the nanopattern of the polymer coatings as well as of the glass plates prior to silane anchor and polymer immobilisation. While PDMAA, PMTA and uncoated glass plates showed similar R_a_ values (0.4 nm ± 0.17, 0.66 nm ± 0.14 and 0.62 nm ± 0.13, respectively), the roughness of PEtOx (1.82 nm ± 1.4) was found to be significantly higher. As it can be observed in Fig 1A–1C, all polymer-coated glass surfaces showed indentations with sizes varying from few nanometers to some micrometers. On PDMAA, small deepenings with depths of approximately 4 nm and widths of about 0.1–0.2 μm were observed (Fig 1A, cross section). The PMTA monolayer revealed similar features with same widths, but lower depths ranging from 1.5 to 2.0 nm (Fig 1C, cross section). As well, glass surfaces without coating showed nano-indentions with depths of around 1 nm, but larger widths of 0.5–1 μm (Fig 1D, white arrows and cross section). In contrast to PDMAA, PMTA and glass surfaces the hollows on PEtOx were found to be much larger both in width and depth (Fig 1B). The roundly shaped indentations demonstrated diameters of approximately 2 μm and depths of around 50 nm (Fig 1B, cross section). Moreover, long channel-like structures with depths of 10–12 nm and widths of 2 μm passing through the whole PEtOx coating could be also observed (Fig 1B). As shown in Fig 1B–1D additional scratch-like structures were detected on both PEtOx and PMTA layers as well on uncoated glass surfaces. However, those were not found on PDMAA layers (Fig 1A).

### Characterisation of the cells of the SG adhering on PMTA, PDMAA and PEtOx

**Fig 2:**
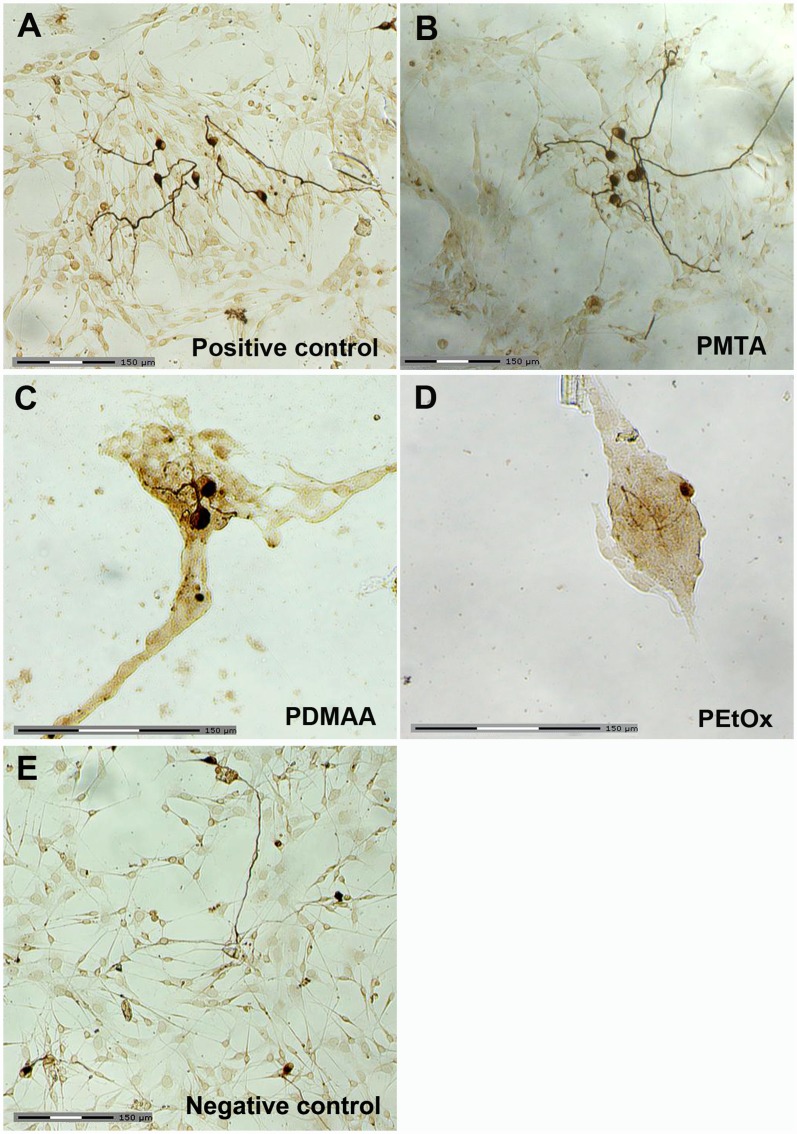
Representative images of DAB stained SGN adhering on polymer-coated glass plates following 48 h cultivation. Representative transmission light microscopic view of the SGN and their neuritogenesis following cultivation on (**A**) positive control (glass plates with ornithine/laminin coating alone), (**B**) PMTA, (**C**) PDMAA, (**D**) PEtOx and (**E**) negative control (glass plate without any coating). The SGN were incubated with the mouse anti-neurofilament antibody and stained with diaminobenzidine (DAB) via binding with the biotinylated anti-mouse IgG-antibody and ABC complex. Scale bars indicate 150 μm.

As first, SGN positively stained with the anti-neurofilament antibody could be demonstrated on the polymer films, positive (glass plates coated with ornithine/laminin) as well as negative control (glass plates without any coating). Both PMTA monolayer and the positive control allowed nearly widespread adhesion and growth of both neurons and non-neuronal cells (Fig 2A and 2B). In contrast, PDMAA and PEtOx surfaces strongly restricted cell attachment (Fig 2C and 2D), whereas glass plates without any coatings induced moderate cell adhesion as shown in Fig 2E. Furthermore, formation of islets of dense packaged and partially shrunk non-neuronal cells on both PDMAA and PEtOx surfaces were observed. Also, filopodia and lamellopodia of the non-neuronal cells could be detected. In contrast, the morphology of the SGN, which were embedded on their non-neuronal cells appeared normal and differentiated, even though they evidently presented shorter neurites in comparison to those adhering on PMTA and the positive control (Fig 2C and 2D).

**Fig 3:**
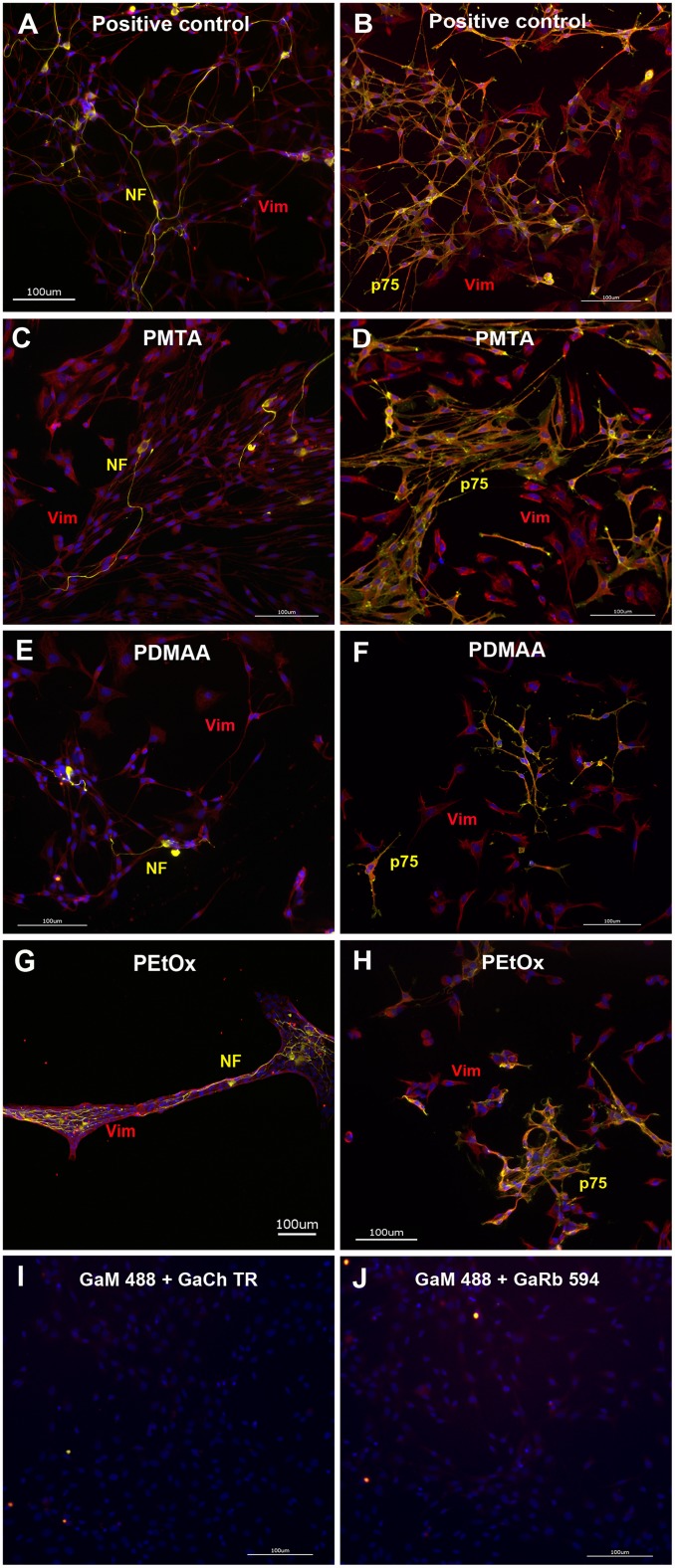
Representative fluorescence images of immunostained SG cells following 48 h cultivation on polymer coated glass plates. Representative fluorescent microscopic views of SGN and non-neuronal cells double labelled either with anti-neurofilament (NF, yellow) and anti-Vimentin (Vim, red) antibody or anti-p75 neurotrophic growth factor receptor (p75-NGFR, assigned in images as p75, yellow) and anti-Vim antibody. Nuclei were stained with DAPI. Positive staining against neurofilament and vimentin antigens were found in cells adhering on glass plates (**A**), PMTA (**C**), PDMAA (**E**) and PEtOx (**G**) demonstrating specific staining of SGN as well as fibroblasts and glial cells. To distinguish glial cells from fibroblasts, the glial cell specific anti-p75-NGFR antibody was used. Expression of p75-NGFR could be shown for glial cells adhering on glass plates (**B**), PMTA (**D**), PDMAA (**F**) and PEtOx (**H**). Images (**I**) and (**J**) represent assays incubated with the secondary antibodies goat anti-mouse conjugated with Alexa Fluor 488 (GaM 488), goat anti-chicken conjugated with Texas Red (GaCh TR) and goat anti-rabbit conjugated with Alexa Fluor 594 (GaRb 594) in the absence of the primary antibodies to demonstrate the specificity of the immune staining. Within the light exposure range between 1.5 and 2 sec no crossreactions between antigens and secondary antibodies as well among both secondary antibodies used for double staining were found.

To verify the composition of the cell population we could demonstrate the presence of the neurofilament antigen exclusively expressed in SGN as well of the intermediary filament vimentin expressed in both fibroblasts and glial cells on all polymer layers (Fig 3A, 3C, 3E and 3G). The adhesion of glial cells on PMTA, PDMAA and PEtOx could be verified by their double staining with anti-vimentin and glial cell specific anti-p75 NGFR antibody (Fig 3B, 3D, 3F and 3H). We found a widespread distribution of all cell types on both PMTA surfaces and the positive control, whereas fibroblasts revealed predominant presence on PDMAA and PEtOx surfaces in comparison to glial cells (Fig 3F and 3H).

**Fig 4:**
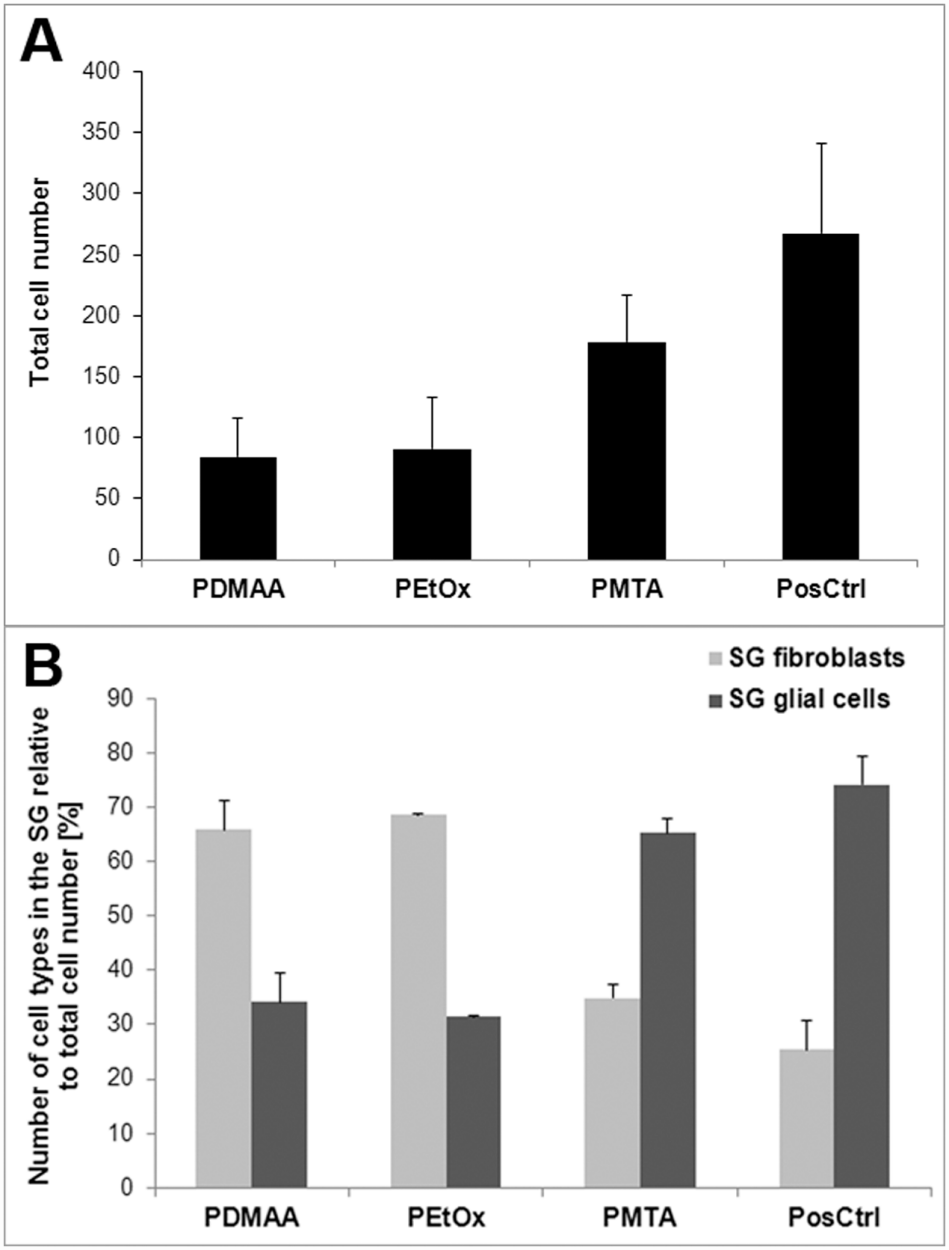
Determination of the number of fibroblasts and glial cells cultivated on polymer coated and uncoated glass plates following immunostaining in relation to the total cell number. Cell count of the fibroblasts and glial cells presented in SG following positive staining with anti-vimentin and anti-p75 antibodies in relation to the total number of DAPI stained cells. The cells were seeded on glass plates with polymer layers PDMAA, PMTA and PEtOx as well on glass plates with ornithine/laminin coating assigned as positive control (PosCtrl). Each data point is presented as mean and SEM of the total cell number (**A**) and percentage of the number of fibroblasts and glial cells in relation to the total cell number counted in n = 2 (PEtOx), n = 4 (PosCtrl.) and n = 5 (PDMAA, PMTA) fluorescence images (**B**).

To verify the assumption that not only neuronal, but also glial cell attachment was strongly inhibited on both PDMAA and PEtOx films, the number of fibroblasts and glial cells were related to the total number of DAPI stained cell nuclei. As shown in Fig 4A the total DAPI stained cell population decreased strongly on the PDMAA and PEtOx monolayers in comparison to those adhering on the positive control, whereas cell attachment on the PMTA surface appeared only slightly restricted. Interestingly, as presented in Fig 4B glial cells were predominant on both PMTA (65.14% ± 2.60) and the positive control (74.05% ± 5.31), whereas the number of fibroblasts in relation to the total cell number was clearly limited on both surfaces (34.86% ± 2.60 and 25.42% ± 5.40). Therefore, not only neuronal, but also glial cell attachment was strongly inhibited on both PDMAA and PEtOx films.

**Fig 5:**
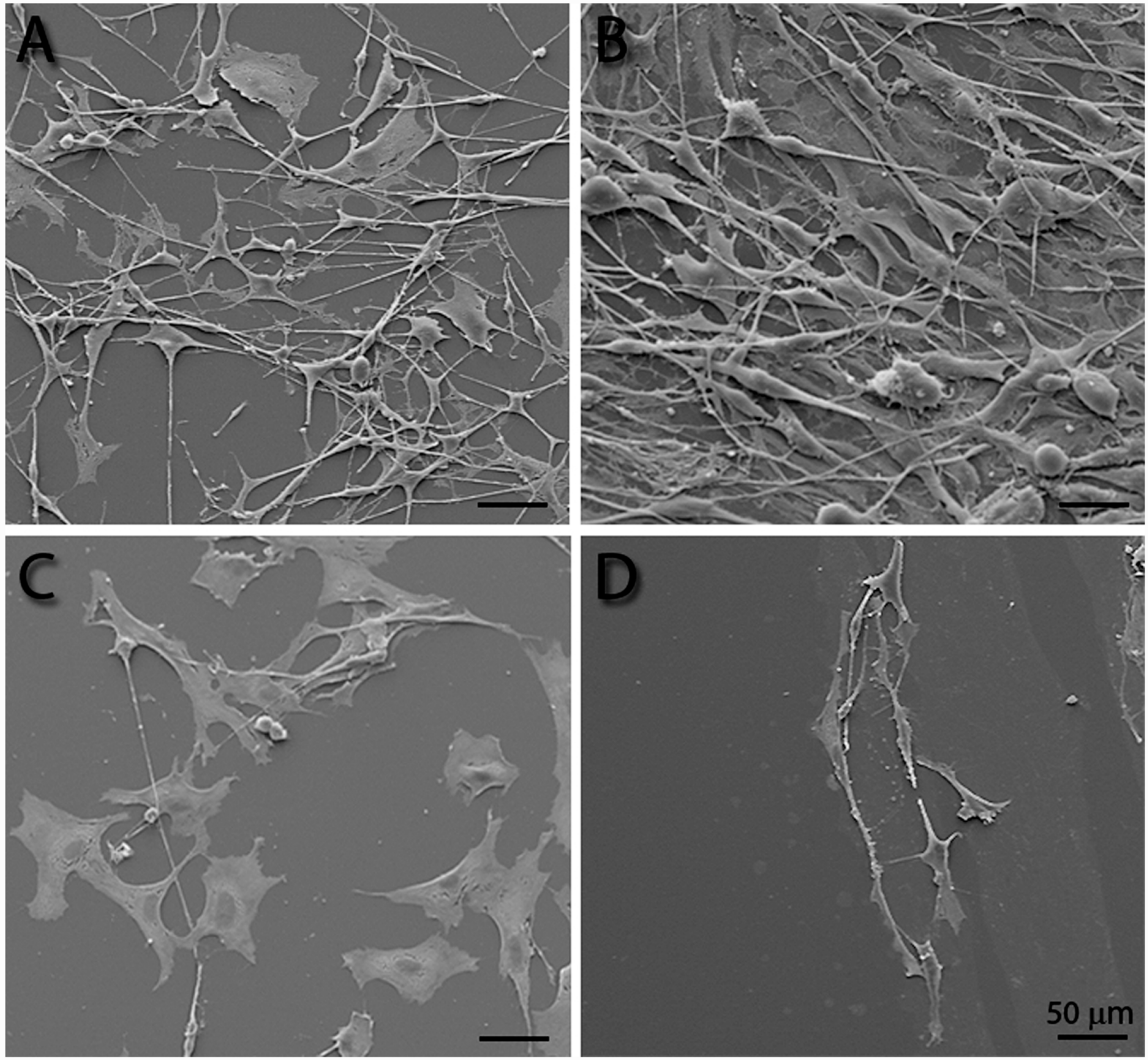
Scanning electron microsopy of the adherent SGN following cultivation on polymer monolayers and ornithine/laminin coated glass plates. Two days after seeding all cell types of the SG were distributed on ornithine/laminin coated glass plates showing characteristic morphologies (positive control, **A**) as well on PMTA layers (**B**), whereas on PEtOx films (**C**) flat cells with broad lamellopodia were mainly observed. In contrast, only few cells were adherent on the PDMAA coated surfaces (**D**). Tiny spherical spots with different sizes in the range of less than 1 μm were visible on both PEtOx (**C**) and PDMAA coatings (**D**). They indicate either adsorption of ECM molecules or residual cell bodies or extensions, which were putatively torn off due to lacking mechanical stability. Furthermore, flattened pale spots could be exclusively observed on PEtOx coatings (**C**) indicating attempts of the cells to form adhesion points due to areas without polymer coating.

Additionally, SEM was used to achieve a closer view of morphological changes of the SGN and non-neuronal cells following 48 h cultivation on polymer coated and uncoated glass plates. As presented in Fig 5A and 5B, all SG cell types retained their morphological characteristics only on the PMTA coatings as well on the positive controls (Fig 5A and 5B). The neuronal outgrowth allowed intensive contacts to the non-neuronal cells forming a dense network of lamello- and filipodia and neurites for trophic support. In contrast, only flat fibroblast like cells were present on PEtOx films tightly connected by expanded lamellopodia (Fig 5C) and the PDMAA surface revealed anti-adhesive effects sweeping away all cell types in the culture (Fig 5D). Additionally, tiny spherical spots with different sizes in the range of less than 1 μm were visible on both PEtOx and PDMAA coatings (Fig 5C and 5D). Furthermore, a second type of flattened pale areas could be observed exclusively on PEtOx coatings.

### SG neuron survival rate and neurite outgrowth were significantly decreased on PDMAA and PEtOx coatings

**Fig 6:**
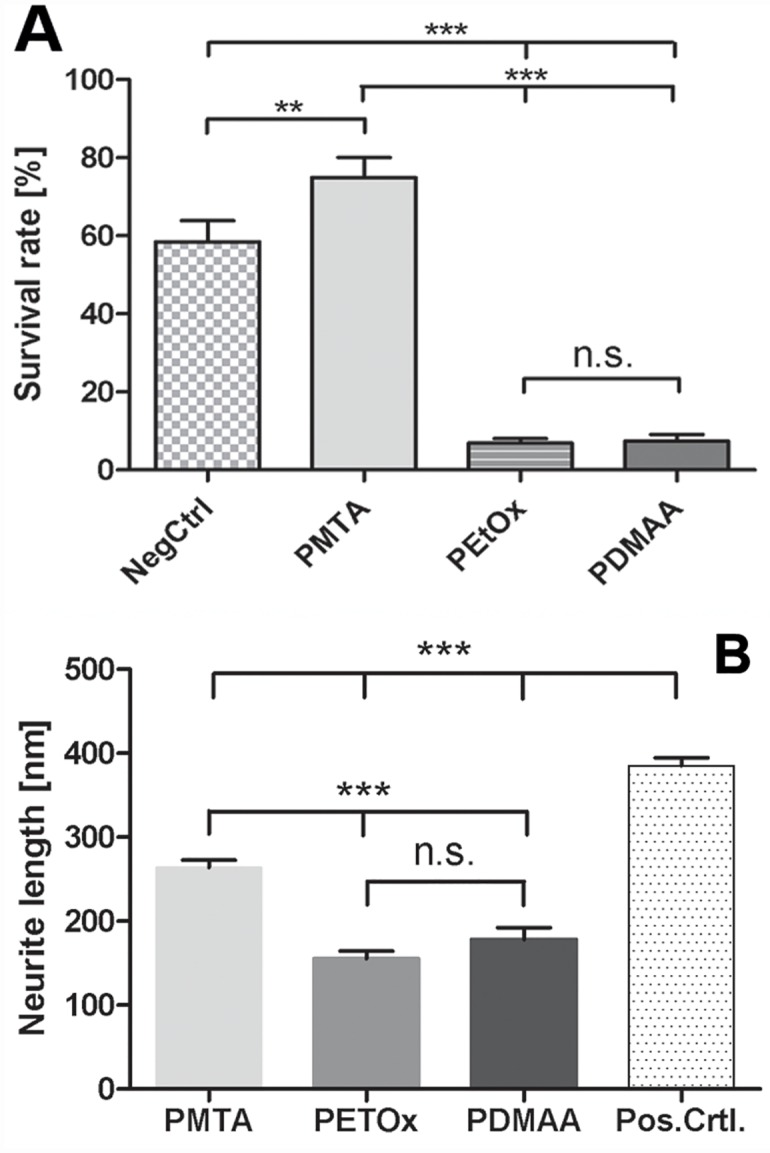
Determination of the survival and neuritogenesis of SGN seeded on polymer-coated glass plates. Effects of polymer (PMTA, PEtOx and PDMAA) coated glass plates on (**A**) survival and (**B**) neurite outgrowth of SGN following 48 h cultivation. Each data point is presented as mean and SEM of the (**A**) percentage of stained SGN soma adhering on polymer coated plates (n = 25 of each polymer coated glass plates) in relation to the positive control (PosCtrl, ornithine/laminin coated glass plates, n = 25) and (**B**) length of nerve fibres (n = 125 neurons sprouted on PDMAA, PEtOx, PMTA and PosCtrl., respectively). ANOVA with Newman-Keuls multiple comparison test was used for statistical assessment (ns, not significant, **p ≤ 0.01, ***p ≤ 0.001).

To characterise the interplay of the SGN with the respective polymer surface, the survival rate of the SGN and their ability to induce neurite growth were quantitatively determined following 48 h of cultivation. As presented in Fig 6A, the number of surviving SGN was significantly restricted on PDMAA and PEtOx: In comparison to the positive control, less than 10% of the neurons were found on PDMAA (7.4% ± 1.71) and PEtOx (6.88% ± 1.15). In contrast, PMTA (74.91% ± 5.11) showed significant adhesive effects on neurons, fibroblasts and glial cells of the spiral ganglion (Fig 6A). When compared to the positive control cell attachment on PMTA was slightly, but not significant, restricted. Survival of SGN was found to be significantly inhibited on glass plates (58.46% ± 5.38) in comparison to the positive control and PMTA. However, attachment of SGN on PMTA was significantly increased in comparison to those on PDMAA and PEtOx (Fig 6A).

In congruence with the survival rate results, we found significant neurite outgrowth in SGN adhering on PMTA (263.8 nm ± 8.82), whereas only poor axonal formation was obtained on both PDMAA (178.3 nm ± 13.83) and PEtOx (155.3 nm ± 8.85, Fig 6B). However, neurite extension on PMTA was significantly lower than on the positive control (385.0 nm ± 9.30). The neuronal outgrowth of the negative control was not considered, since its average neurite lengths could be assumed within the range of those found on PMTA and positive control.

## Discussion

The growth of intracochlear tissue following the insertion of the electrode array is thought to be the result of an inflammatory foreign body reaction, the contamination of perilymph with blood or bone dust, or a local infection following surgery [54–56]. In consequence the impedance at electrode–tissue interface increases [13–14] and the selective neuronal stimulation for discrimination between different sound frequencies is disturbed. Thus, future strategies aim at preventing fibrotic tissue formation and encapsulation of the electrode contacts either by pharmacological impact, chemical or topographical modification of the carrier material. Implant-based local drug delivery without systemic toxic side-effects is an attractive therapeutic approach to control such cellular processes. Pharmacological substances as like mytomycin C, fluorouracil-5, paclitaxel [28] and dexamethasone [57–61] were described as anti-proliferative, anti-migrating and anti-inflammatory agents. Other studies investigated topographically modified biomedical material surfaces in the micro- and nanometer scale to study their impact on the cell behaviour *in vitro* [62–68]. Beside controlling the topography for minimizing cell adhesion chemical surface modifications may be another approach to gain cell selective implant surfaces. So far, the anti-adhesive and–proliferative impact of PDMAA and PEtOx coatings had been demonstrated on the murine fibroblast cell line NIH 3T3 as a cell model for connective tissue [32, 35]. Here, we present for the first time their effects on primary cells of the inner ear of postnatal rats comprising SGN, fibroblasts and glial cells.

### Characterisation of the polymer coating

Since homogeneity of the attached monolayer may have a strong impact on surface properties and subsequent cell attachment [40], WCA measurements, ellipsometry and AFM were used to characterise both the silane anchor and the overlaying polymer films. The observed rise of the WCA and the measured layer thickness for the glass plates functionalized with the silane anchor are in good agreement with previously reported data and indicate a consistent coverage of the glass surface with a monolayer of anchor molecules [35, 50, 69]. The WCAs obtained for the coating with PMTA (16°), PDMAA (33°) and PEtOx (42°) were found to be in accordance with those reported earlier and allow the conclusion, that all polymer films could be considered as hydrophilic [31, 35, 40]. The ellipsometric detected layer thickness for the three polymers ranging between 5 nm (PMTA) and 12 nm (PEtOx) confirms the presence of ultrathin films on the silane anchor monolayer and is in good accordance with already demonstrated results of other studies [31–32, 35]. It should be noted, that the resulting layer thickness is depending on the molecular weight or, more precisely, on the radius of gyration of the respective polymer attached to the silane anchor [50]. Thus, differences between the observed layer thicknesses in our investigations and the measured thicknesses in other studies may be caused by different molecular weights of the used polymers. Moreover, inhomogeneities and defects in the polymer coatings affect the ellipsometric measurement of the layer thickness and cause inaccuracies in the obtained values. An Incomplete coverage of the substrates also influence the resulting contact angles determined for the polymer coatings and may explain the discrepancies for the obtained contact angles in the different studies.

However, WCA and ellipsometric measurements cannot give any informations about the nanometer scaled surface roughness. Only imaging of the surface topography by AFM revealed highly rough surfaces of the PEtOx monolayer in comparison to those of the PDMAA and PMTA films and glass plates (Fig 1, Table 1). The indentations with varying widths and depths found on all polymer surfaces imply incomplete or inhomogeneous coverage of the glass plate with either the anchor molecules or the polymer film as also reported earlier by Aliuos et al. 2014 [35]: Hereby, AFM-based single-cell force spectroscopy was used to measure the adhesion forces of single 3T3 NIH cells adhering on PDMAA and PEtOx coatings. It was found that indentions in the nanometer scale on both PDMAA and PEtOx due to uncoated anchor molecules may have allowed adsorption of ECM molecules. In the consequence formation of the ECM facilitates few adhesion points between the glass surfaces and cells leading to small initial cell adhesion forces [35]. Moreover, it was found that uncoated anchor molecules demonstrated even stronger adhesion effects rather than glass [35]. These conclusions seem to match our observations of formation of cell islets comprising mainly fibroblasts on PEtOx and PDMAA films facilitated by those cavities (Figs 2C and 2D and 3E–3H). Especially SEM images show preferential adhesion of fibroblasts on these polymers. The well spread fibroblasts with expanding lamellopodia indicate successful cell adhesion onto the polymer surfaces. It is suggested that the cells used the nano-scaled indentions for formation of adhesion points and to attach to the anti-adhesive PDMAA- and PEtOx-coated surfaces.

Additionally, tiny spherical spots with different sizes detected on both PDMAA and PEtOx coatings indicate either adsorption of ECM molecules or residual cell bodies, which were putatively torn off during processing of the cells for electron microscopy due to lacking mechanical stability. Otherwise, it can also be speculated that those spots present areas of protein adsorption from the culture medium containing serum proteins and neurotrophic factors. The flattened pale spots observed on PEtOx only correspond well with the findings by using AFM: the form and size of these areas show similarity with the indentations on PEtOx surfaces found on AFM images (Fig 1B, upper and lower row). As discussed above, they are suggested to attribute to incompletely coated surface areas.

Beside the round-shaped indentions channel-like deepenings with widths of around 2 μm and depths of 10–12 nm were detected on untreated glass, PEtOx and PMTA films indicating scratches on the glass surfaces. Hereby, silane anchor attachment with subsequent photochemical binding of the polymers may part wise trace the topography of the glass surface pretending a patchy surface coating.

### Interactions between the polymer monolayers and SGN and non-neuronal cells

PMTA (16° ± 5) showed the lowest WCA value in comparison to the polymers PDMAA (33° ± 1) and PEtOx (42° ± 1) indicating the most hydrophilic and, thus, anti-adhesive surface as described in previous studies [25, 70–71]. However, the high number of adhering SGN cultivated on PMTA coated glass plates as well as the significant increase of the average neurite length in comparison to those on PDMAA and PEtOx coatings were in contradiction to this conclusion. Also, the counting of non-neuronal cells revealed an increase of cell growth on PMTA, but not on PDMAA and PEtOx. Indeed, no clear correlation between the physico-chemical properties of the polymer monolayers and their suitability as a substrate for cell growth independently of the cell type could be found so far [40, 31–32, 37]. Adden et al., 2007, concluded that a WCA higher than 45° was necessary for a high growth score of osteogenic precursor cells, but the interaction between cells and the polymer monolayer could not be related to the water contact angle only [32]. Hereby, cell adhesion on PDMAA, PEtOx and PMTA may relate to the interaction of the cell membrane of the respective cell type either with the electrical charge or with the chemical groups of the polymer layers. In example the swelling capacity of the polymer in aqueous solutions influences the cell behaviour as it was demonstrated for PDMAA and PEtOx so far [34, 72].

Considering the interaction of the cell membrane with the electrical charge, it seems very likely that PMTA provides a good substrate for adhesion of the SGN and its non-neuronal cells due to its high positive charge density [31, 37, 39, 40, 73]. Especially neuronal cells may be responsive to positively charged polymer surfaces: It was reported that *oligo-(polyethylene glycol) fumarate* (OPF) modified with PMTA monomer supported adhesion and differentiation of dorsal root ganglion neurons in a dose-dependent manner [48]. As reviewed by Bacakova et al., 2011 [25], the reason for better cell adhesion to positively charged surfaces is the presence of negatively charged ECM molecules mediating cell binding. Moreover, positively charged amine groups also act in a synergistic way with oxygen containing groups in stimulating adhesion and growth [25].

Interestingly, PMTA and glass plates with ornithine/laminin coating appeared also as attractive substrates for glial cell adhesion as we have shown for the first time by immunostaining with specific antibodies for neurons, fibroblasts and glial cells. Hereby, the percentage of the number of fibroblasts in relation to the total cell number was clearly scaled down indicating both surfaces as preferential substrates for glial cell attachment and glial cell driven formation of ECM rather than for fibroblasts. In contrast to PMTA, PDMAA and PEtOx represent polar polymers without any positive or negative electrical charge and, thus, are considered as anti-adhesive surfaces [74–75]. In comparison to those on PMTA and the positive control, we found not only strongly restricted adhesion of SGN as well as of the non-neuronal cells on PEtOx and PDMAA layers, but also significantly shorter neurites (Figs 2–4 and 6). Hereby, single spherical cells as well islets of partially retracted non-neuronal cells were observed on PDMAA and PEtOx indicating changings of the cell morphology due to the interaction with the polymers. As also shown by SEM imaging both polymer surfaces impeded adhesion of glial cells and consequently the survival of neurons. Only fibroblasts with broad lamellopodia were mainly detected on PEtOx layers indicating the formation of adhesion points due to nano-scaled indentions of the polymer layer as discussed above.

However, the relationship of the number of glial cells growing on the PEtOx and PDMAA surfaces to those of the fibroblasts was reversed. This finding indicates that polarity or electric charge of polymer layers among other parameters influence the colonisation of the surfaces with target primary cells. Moreover, we suppose that the reduced SG neuron survival and neuritogenesis following cultivation on PDMAA and PEtOx can be associated with the decrease of the number of supporting glial cells. These cells contribute to mechanisms promoting neuronal survival, growth and regeneration by providing myelination and trophic support and also by expressing cell surface and extracellular matrix proteins [76–77]. They are interconnected by gap junctions which are suggested to be involved in maintenance and protection of the mammal auditory nerve via connexin 43-mediated signaling between SGN [78–79]. Since the glial cells attachment on both PDMAA and PEtOx surfaces was significantly reduced, we conclude, that SG neuron adhesion, survival and neurite extension is strongly associated with the spreading of glial cells on the respective surface. In the consequence, the effects of the polar polymers without any electrical charge may be the result of the repression of the important interaction of SGN and their surrounding glial cells with the chemical groups of the polymer layers. As reported by Whitlon et al., 2009 [77], SGN were strongly involved in molecular interplay with glial cells rather than with the protein coating of the culture dish (laminin, fibronectin or tenascin) or with fibronectin positive cells. In conclusion, our findings clearly show, that the anti-adhesive surfaces of PDMAA and PEtOx inhibit the attachment of glial cells and ECM formation and, thus, impede trophic support and axonal growth of the SGN. In conclusion, the positively charged PMTA present an auspicious polymer coating enabling the adhesion and proliferation of glial cells and indirectly supporting the survival of SGN and their neurite sprout. Further *in vivo* studies need to be conducted to investigate the potential of PMTA as appropriate coating of neurostimulating electrodes to improve the nerve-electrode-interface.

## Abbreviations

AFM: Atomic force microscopy
Ra: average roughness
CI: cochlea implant
ECM: extracellular matrix
PDMAA: *poly(N*,*N-dimethylacrylamide)*
PEtOx: *poly(2-ethyloxazoline)*
PMTA: *poly([2-methacryloyloxy)ethyl]trimethylammoniumchlorid)*
SEM: scanning electron microscopy
SGN: spiral ganglion neurons
WCA: water contact angle
